# Adalimumab-induced pustular psoriasis successfully treated with tofacitinib in a patient with ankylosing spondylitis: A brief report

**DOI:** 10.1016/j.jdcr.2026.08.057

**Published:** 2026-09-21

**Authors:** Duygu Kurtuluş, İlkin Zindancı

**Affiliations:** a Uskudar University Faculty of Medicine, Department of Physical Medicine and Rehabilitation, Memorial Atasehir Hospital, Istanbul, Turkey; b Department of Dermatology, University of Health Sciences, Haydarpaşa Numune Training and Research Hospital, Istanbul, Türkiye

**Keywords:** adalimumab, ankylosing spondylitis, JAK inhibitor, paradoxical psoriasis, pustular psoriasis, tofacitinib

## KEY POINTS

• Severe adalimumab-induced pustular psoriasis may necessitate TNF inhibitor discontinuation in ankylosing spondylitis, creating a major therapeutic dilemma.
• JAK inhibition targets shared inflammatory pathways implicated in both paradoxical psoriasis and axial spondyloarthritis.
• This report provides the first clinical evidence that tofacitinib can achieve simultaneous remission of TNF-induced pustular psoriasis and axial disease activity in ankylosing spondylitis.

## Introduction

Tumor necrosis factor (TNF) inhibitors are widely used in the management of ankylosing spondylitis (AS) and have substantially improved disease control and functional outcomes.^1^ However, paradoxical inflammatory reactions, particularly psoriatic skin manifestations, have been increasingly recognized during anti-TNF therapy.^2^ Among these, pustular psoriasis represents a rare but clinically challenging adverse event, often necessitating treatment discontinuation.^3^ While the underlying mechanisms are not fully elucidated, dysregulation of interferon signaling and downstream activation of the Janus kinase–signal transducer and activator of transcription pathway have been implicated.^4^^,^^5^ Although recent reviews have suggested switching to non–TNF-targeted therapies, including Janus kinase (JAK) inhibitors, as a potential strategy, clinical evidence supporting this approach remains extremely limited.^6^^,^^7^ To the best of our knowledge, no previous reports have described the successful treatment of adalimumab-induced pustular psoriasis with tofacitinib in a patient with AS.

## Case presentation

A 44-year-old male patient with a known diagnosis of AS was followed at our outpatient clinic for several years. He was initially managed with nonsteroidal anti-inflammatory drugs (indomethacin), sulfasalazine, and a structured exercise program. Due to persistently active disease, reflected by a Bath Ankylosing Spondylitis Disease Activity Index score of 6.1 and a Bath Ankylosing Spondylitis Functional Index score of 5.3, adalimumab therapy was initiated at a dose of 40 mg administered subcutaneously every 2 weeks. At treatment initiation, inflammatory markers were within normal limits (erythrocyte sedimentation rate: 18 mm/h; C-reactive protein: 0.3 mg/dL).

After approximately 3 years of continuous adalimumab treatment, the patient developed painful pustular skin lesions involving the hands and feet. Dermatologic evaluation raised suspicion of pustular psoriasis related to anti-TNF therapy. A skin biopsy demonstrated orthokeratotic hyperkeratosis, parakeratosis, intracorneal pustule formation extending into the upper epidermis, focal hypergranulosis, prominent psoriasiform acanthosis, spongiosis, and variable changes in the suprapapillary epidermis, consistent with pustular psoriasis (Fig 1, *A* and *B*). Based on the clinical and histopathological findings, a diagnosis of adalimumab-induced pustular psoriasis was established. Consequently, adalimumab therapy was discontinued, and topical dermatologic treatments were initiated. Representative clinical photographs obtained prior to treatment discontinuation demonstrated widespread pustular lesions affecting the palms and soles (Fig 2, *A*-*C*).

**Fig 1:**
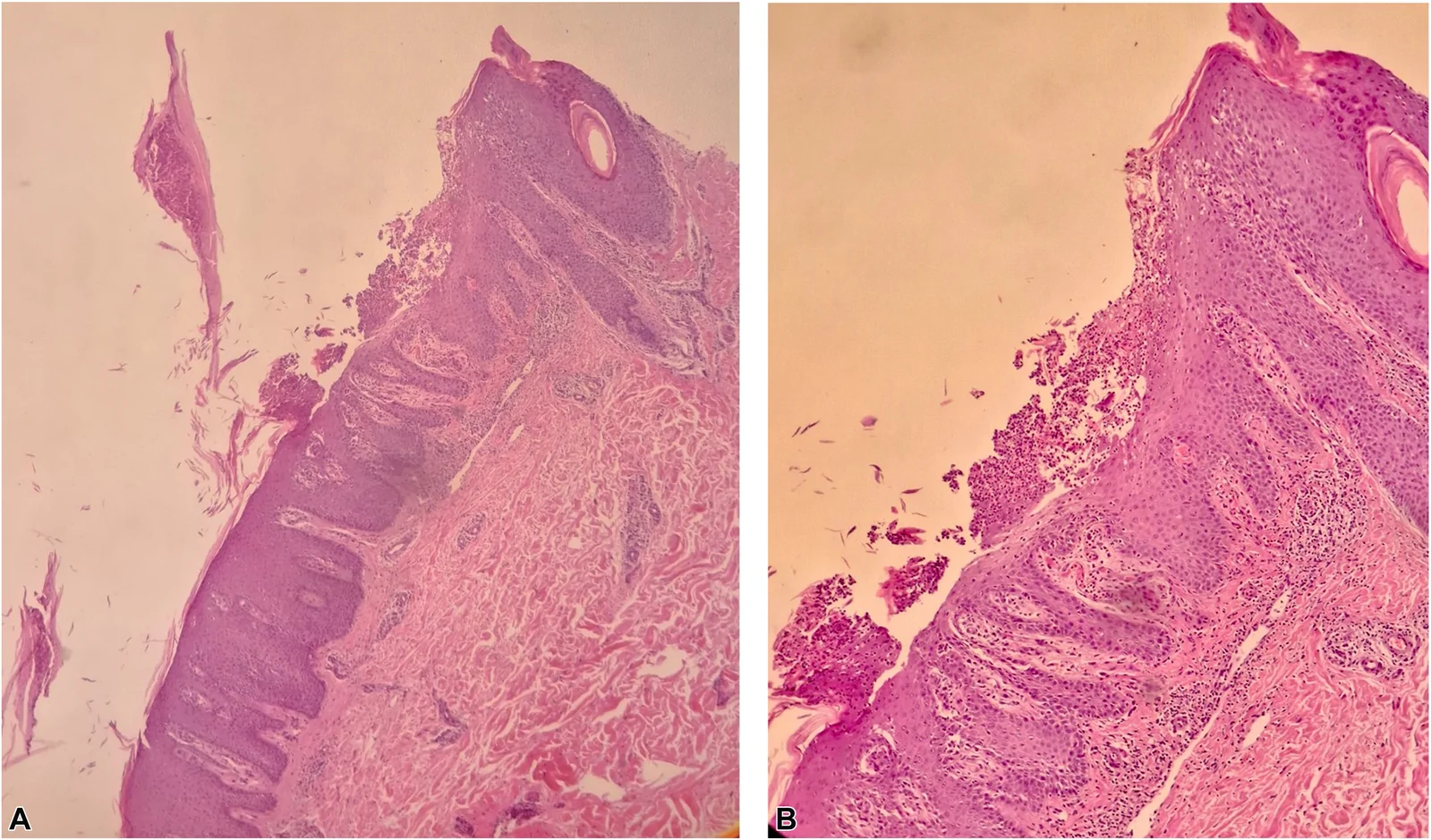
Histopathological findings of pustular psoriasis. **A,** Low-power view demonstrating orthokeratotic hyperkeratosis, parakeratosis, and intracorneal pustule formation extending into the upper epidermis (H&E, ×40). **B,** Higher-power view showing an intracorneal pustule extending into the upper epidermis, with focal hypergranulosis, prominent psoriasiform acanthosis, spongiosis, and variable thinning and thickening of the suprapapillary epidermis (H&E, ×100).

**Fig 2:**
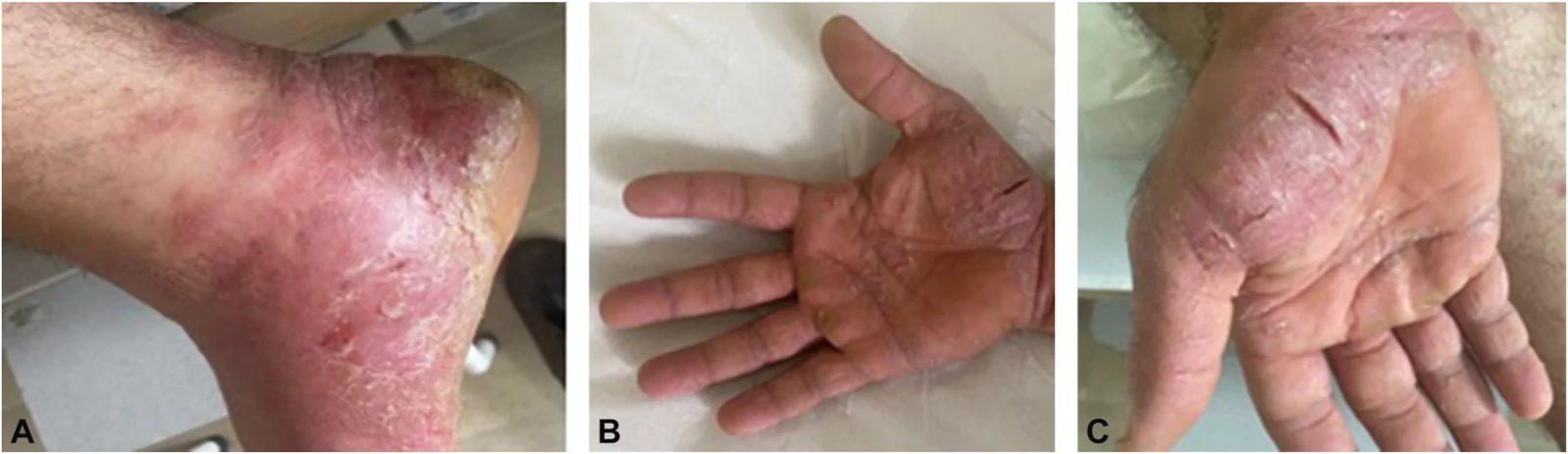
**A-C,** Representative clinical photographs obtained prior to treatment discontinuation demonstrated erythematous, hyperkeratotic plaques with scaling, fissuring, and pustular lesions involving the palms and periarticular regions of the hand.

Following discontinuation of adalimumab, the patient was managed with nonsteroidal anti-inflammatory drugs and sulfasalazine; however, his axial symptoms progressively worsened. Several months later, he re-presented with severe inflammatory back pain, widespread musculoskeletal pain, and marked functional limitation. Disease activity assessment demonstrated a Bath Ankylosing Spondylitis Disease Activity Index score of 7.3, indicating high disease activity. Given the history of anti-TNF–induced pustular psoriasis and the need for effective control of both articular and extra-articular manifestations, treatment with tofacitinib was initiated at a dose of 11 mg once daily.

Within 1 month of initiating tofacitinib therapy, the patient reported marked improvement in axial pain and functional capacity, accompanied by substantial regression of the pustular skin lesions. This clinical improvement was paralleled by normalization of inflammatory markers at follow-up. At subsequent visits, both musculoskeletal symptoms and dermatologic findings continued to improve without the need for additional systemic therapy. Follow-up clinical photographs demonstrated near-complete resolution of the pustular lesions (Fig 3, *A* and *B*). At the time of writing, the patient has been receiving tofacitinib for 6 months and remains in sustained clinical remission with no recurrence of cutaneous lesions.

**Fig 3:**
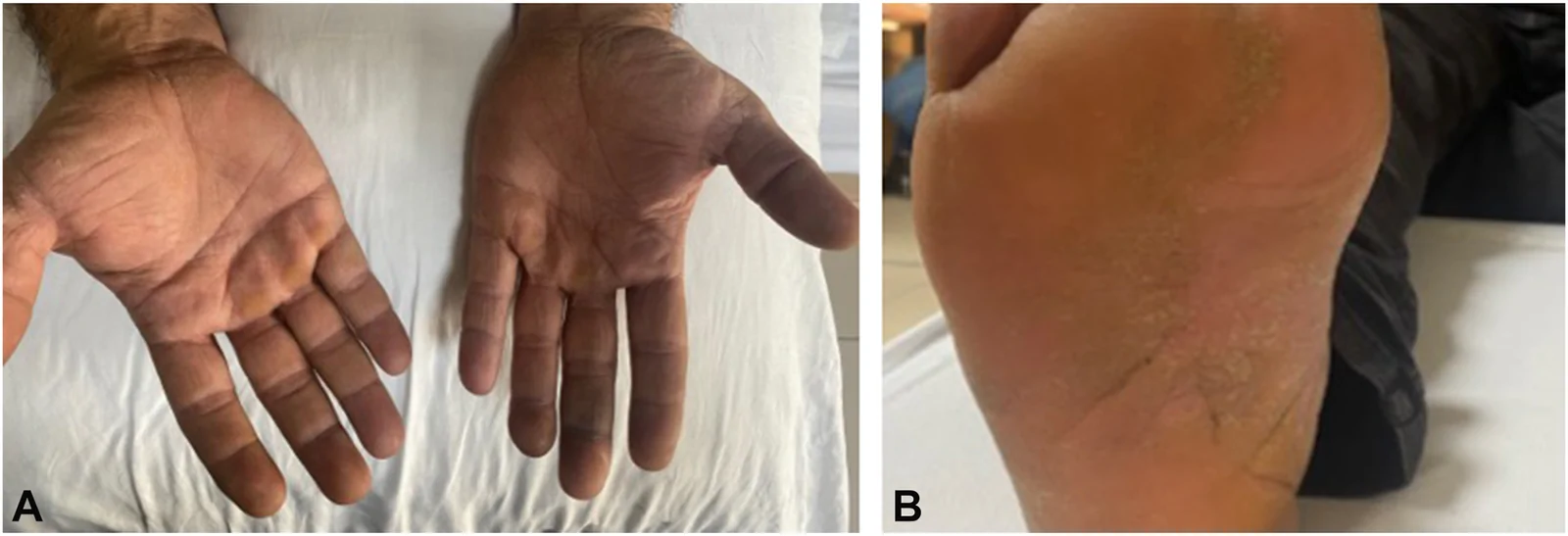
Clinical improvement following tofacitinib therapy. **A,** Near-complete resolution of palmoplantar lesions with normalization of skin texture and absence of pustules after treatment. **B,** Marked improvement of plantar involvement with resolution of hyperkeratosis, fissuring, and inflammatory changes.

The patient provided written informed consent for the publication of this case report and any accompanying images.

## Discussion

Paradoxical psoriatic reactions are a well-recognized but uncommon adverse effect of TNF inhibitor therapy and have been increasingly reported in patients with spondyloarthritis.^2^^,^^6^^,^^8^^,^^9^ Although the exact pathophysiological mechanisms remain incompletely understood, accumulating evidence suggests that TNF blockade may lead to dysregulated interferon signaling, activation of plasmacytoid dendritic cells, and downstream stimulation of the Janus kinase–signal transducer and activator of transcription pathway.^6^^,^^10^^,^^11^ Pustular psoriasis represents one of the more severe phenotypes within this spectrum and often necessitates discontinuation of the offending agent.^3^^,^^4^ In patients with AS, management of paradoxical psoriasis is particularly challenging, as withdrawal of TNF inhibition may result in loss of disease control and limited alternative therapeutic options.^1^

Recent reviews have suggested that switching to non–TNF-targeted therapies, such as interleukin 17 or Janus JAK inhibitors, may represent a rational strategy for managing severe paradoxical psoriasis in patients with AS; however, clinical evidence supporting this approach remains scarce.^2^^,^^5^ Although interleukin 17 inhibitors are effective options for axial spondyloarthritis,^1^ their role in paradoxical pustular psoriasis remains less clearly defined, and concerns regarding variable cutaneous responses may limit their use in certain clinical scenarios.^4^^,^^12^

In this context, our case provides real-world clinical support for the theoretical rationale underlying JAK inhibition, demonstrating successful control of both cutaneous and axial manifestations following initiation of tofacitinib.^7^^,^^13^^,^^14^ The dual efficacy observed in our patient may be explained by the central role of the Janus kinase–signal transducer and activator of transcription pathway in mediating interferon-driven inflammation implicated in paradoxical psoriasis, while simultaneously modulating inflammatory signaling involved in axial spondyloarthritis.^5^^,^^10^^,^^13^ To the best of our knowledge, this is the first reported case describing the successful use of tofacitinib for adalimumab-induced pustular psoriasis in a patient with AS.

Although conclusions cannot be generalized from a single case, our findings suggest that JAK inhibitors may represent a promising therapeutic alternative in selected patients who develop severe dermatologic adverse events during TNF inhibitor therapy.^5^^,^^7^^,^^15^ In this context, JAK inhibition may provide simultaneous control of both cutaneous and axial manifestations following anti-TNF discontinuation, supporting its mechanistic rationale in patients with AS complicated by paradoxical pustular psoriasis. Further studies are warranted to clarify the role of JAK inhibitors in managing paradoxical inflammatory reactions associated with TNF blockade.

## Conflicts of interest

None disclosed.
